# Gastrointestinal disorders and intestinal bacteria: Advances in research and applications in therapy

**DOI:** 10.3389/fmed.2022.935676

**Published:** 2023-02-07

**Authors:** Toshifumi Ohkusa, Yuriko Nishikawa, Nobuhiro Sato

**Affiliations:** Department of Microbiota Research, Juntendo University Graduate School of Medicine, Tokyo, Japan

**Keywords:** gut microbiota, inflammatory bowel disease, NSAID enteritis, colorectal cancer, alcoholic and non-alcoholic steatohepatitis, primary sclerosing cholangitis, pancreatic cancer, fecal microbiota transplantation

## Abstract

Intestinal bacteria coexist with humans and play a role in suppressing the invasion of pathogens, producing short-chain fatty acids, producing vitamins, and controlling the immune system. Studies have been carried out on culturable bacterial species using bacterial culture methods for many years. However, as metagenomic analysis of bacterial genes has been developed since the 1990s, it has recently revealed that many bacteria in the intestine cannot be cultured and that approximately 1,000 species and 40 trillion bacteria are present in the gut microbiota. Furthermore, the composition of the microbiota is different in each disease state compared with the healthy state, and dysbiosis has received much attention as a cause of various diseases. Regarding gastrointestinal diseases, dysbiosis has been reported to be involved in inflammatory bowel disease, irritable bowel syndrome, and non-alcoholic steatohepatitis. Recent findings have also suggested that dysbiosis is involved in colon cancer, liver cancer, pancreatic cancer, esophageal cancer, and so on. This review focuses on the relationship between the gut microbiota and gastrointestinal/hepatobiliary diseases and also discusses new therapies targeting the gut microbiota.

## 1. Introduction

Since the 1990s, direct sequencing targeting bacterial-specific 16S ribosomal RNA (16S rRNA) and 16S rRNA-encoding genes have been adopted to study intestinal bacteria, which has enabled the identification of bacterial species that have remained uncultured. In addition, the emergence of next-generation sequencing technologies has accelerated the gene analysis process and led to metagenomic analysis, which contributed to the elucidation of enormous quantities of gut microbiota members. Metagenomic shotgun sequencing and 16S amplicon sequencing are commonly used for the analysis of gut microbiota. Although each of these two types of analysis has advantages and disadvantages (Table 1), both have brought about great advances in gut microbiome analysis. Such advances in bacterial analyses have shed light on dysbiosis, i.e., altered microbiota compositions in individuals with diseases compared with the microbiota compositions of healthy individuals. An association of this perturbed state of the gut microbiota with various diseases has been reported to occur in inflammatory bowel disease (IBD), irritable bowel syndrome (IBS), non-alcoholic steatohepatitis (NASH), and colorectal, pancreatic, and esophageal cancers. The association with additional disorders has also been implicated, including diabetes, obesity, arteriosclerosis, multiple sclerosis, neuropsychiatric conditions (such as autism), uremia, and rheumatoid arthritis. In this study, the relationships between intestinal bacteria and individual gastrointestinal (GI) diseases that have been reported to be associated with intestinal microbiota dysbiosis (Table 2) are discussed.

**TABLE 1:** Commonly used methods to assess the microbiota.

Method	16S amplicon sequencing	Shotgun sequencing
Cost	Less expensive	Expensive
Detection	Detecting taxa in low abundance	Miss taxa present in low abundance
Identifying species and genes	Less reliable	More useful
Functional relevant	Not available	Available

**TABLE 2:** GI system diseases reported to be associated with intestinal microbiota dysbiosis.

GI diseases
IBD IBS
Antibiotic-associated colitis (*Clostridium difficile* colitis, CDI)
Colorectal cancer
Esophageal cancer
Non-steroidal anti-inflammatory drug-induced colitis
Hepatic diseases
Alcoholic steatohepatitis (ASH)
Non-alcoholic steatohepatitis (NASH)
Liver cirrhosis (LC)
Hepatic encephalopathy (HE)
Primary sclerosing cholangitis (PSC)
Pancreatic disease
Pancreatic cancer

## 2. IBD and intestinal bacteria

The pathogenesis of intestinal inflammation in IBD was previously attributed to autoimmunity (autoimmunity theory). However, due to advances in research through the development of bacterial metagenomic analysis methods and the discovery of innate immunity, intestinal bacteria have surfaced as a culprit in IBD, and the supporting data are presented in Table 3.

**TABLE 3:** Supporting data for the theory of intestinal bacteria as a cause of IBD.

Supporting data
□ Immune abnormality-related spontaneous enterocolitis does not develop in germ-free settings; it is caused by intestinal bacteria
□ Resident bacteria are extremely abundant in the intestinal mucosa of patients with IBD
□ The beneficial vs. harmful bacterial balance is abnormal with harmful species predominatingin patients with IBD compared with the balance in healthy individuals
□ Genetic polymorphism analysis reveals that many of the gene polymorphisms observed in IBD are linked to the reduction in bacterial clearance and mucosal protective mechanisms
□ In IBD, tolerance to intestinal bacteria decreases, resulting in an excessive immune response, i.e., inflammation
□ Toll-like receptors, key players in the innate immune system, are expressed in the intestinalepithelium, and for many of them, the ligands are bacterial components.

### 2.1. Intestinal mucosal protection capacity declines in IBD due to leaky gut

In the research of single-nucleotide polymorphisms (SNPs) in IBD, the availability of further advanced sequencing technologies has enabled genome-wide association studies (GWASs), in which more and longer gene sequences are simultaneously analyzed. Using such sequencing systems, 71 Crohn’s disease (CD)-associated SNPs and 47 ulcerative colitis (UC)-associated SNPs were identified in the European and American populations studied (1, 2). For approximately 80% of those SNPs, their roles are not clear, but among the remaining SNPs, many are known to have roles related to bacterial clearance or mucosal protection (3). NOD2 (CARD15), a particularly well-known CD-associated gene predominantly expressed in the Paneth cells of the small intestine, detects the bacterial component peptidoglycan through the recognition of the peptidoglycan product muramyl dipeptide (4). Moreover, NOD2 induces defensin secretion by Paneth cells for bacterial elimination (5). NOD2 also promotes the production of immunosuppressive interleukin-10 (IL-10) (6) and is involved in the induction of autophagy, a recent high-profile topic (7, 8). With respect to autophagy, it has been reported that SNPs in the autophagy-related 16 like 1 (*ATG16L1*) gene are observed in patients with CD and that autophagy is reduced in cells with NOD2 and ATG16L1 variants (7). Given the above, in CD, the function to detect pathogenic bacteria and eliminate them *via* the promotion of defensin secretion assumingly may be diminished, and further, SNPs may hamper the removal of components of invading bacteria from cells; this may actually be a cause of the non-caseating granulomas observed in patients with CD. A recent study detected highly harmful intestinal bacterial counts in the blood of patients with active-phase CD harboring the ATG16L1 variant (9). Another study reported a massive translocation of intestinal microbes into the mesenteric adipose tissue (10). The decreased mucosal defense in genetically influenced patients with CD and pathological bacterial translocation are generally detailed in a recent review (11).

In IBD, especially in UC, attention has been drawn to increased mucosal permeability, or “leaky gut.” In a U.S. multicenter study (3), reduced epithelial-barrier function resulting in the leaky gut was observed in patients with UC carrying SNPs in the following genes: *HNF4A*, which is involved in cell–cell junctions, such as tight junctions and adherens junctions; *CDH1*, an encoder of E-cadherin, which is a protein involved in cell–cell adhesion formation and maintenance; and *LAM*, an encoder of laminin subunit beta-1, which is expressed in the intestinal basement membrane and plays a key role in anchoring the single-layered epithelium. Essentially, one study found a decrease in the levels of the mucin core protein gene *MUC2* and mucus-producing goblet cells in UC-affected mucosae (12). A recent study reported that sialylation plays an essential role in protecting mucus-barrier integrity from bacterial degradation and is governed by ST6GALNAC1 (ST6), a local sialyltransferase in the gut (13). Glycoproteomic profiling and biochemical analysis of ST6 mutations identified in patients show that decreased sialylation causes defective mucus proteins and congenital IBD. In addition, a number of studies have reported the association of a high-fat diet with increased mucosal permeability (14). The possibility of increases in the number of IBD cases in Japan attributable to leaky gut resulting from such a diet cannot be ruled out; specifically, mucosal penetration of commensal bacteria due to a leaky gut may cause intramucosal inflammation or ulceration, leading to the onset of IBD.

Although immune tolerance is important in terms of symbiosis between intestinal bacteria and mucosal cells, multiple GWASs identified SNPs in the immunosuppressive cytokine IL-10, a key factor in immunotolerance, in patients with UC and those with CD, with decreased production of this cytokine in both patient populations studied (1, 15). IL-10-knockout mice spontaneously develop colitis in the presence of intestinal bacteria (16). In humans, it was reported that variants of the IL-10 receptor genes *IL-10RA* and *IL-10RB* caused an early onset of severe IBD (17). These GWAS results were obtained from Western populations. SNPs in NOD2 and ATG16L1 are deemed unlikely in Japanese individuals (18, 19). In addition, evidence for the influence of each SNP on the development of IBD is not considered conclusive, with the odds ratio estimate of the influence being low.

### 2.2. IBD may be caused by gut microbiota dysbiosis

**FIGURE 1:**
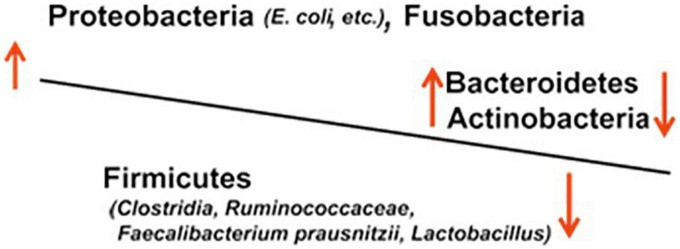
Dysbiosis of the gut microbiota in IBD.

In IBD, gut microbiota dysbiosis, i.e., imbalanced microbiota composition due to decreased anti-inflammatory species and increased pro-inflammatory species, is thought to result in mucosal inflammation and ulceration (Figure 1).

A large number of studies of IBD based on genetic analyses targeting 16S rDNA, which encodes bacterial 16S rRNA, have reported an increase in bacteria of the family Enterobacteriaceae and the genus *Desulfovibrio* of the phylum Proteobacteria, those of the genus *Bacteroides* of the phylum Bacteroidetes, and those of the phylum Fusobacteria (20). The bacteria *Bacteroides* and *Enterobacteriaceae* (e.g., *Escherichia coli* and *Klebsiella*) are pathogens that induce opportunistic infections, such as sepsis, in the compromised host and are classified as aggressive microbial species, i.e., harmful species. On the contrary, a number of studies in patients with UC or CD have reported a decrease in beneficial microbial species or probiotics, including *Lactobacillus, Faecalibacterium prausnitzii, Roseburia hominis*, and *Clostridium* IXa and IV groups of the phylum Firmicutes and genus *Bifidobacterium* of the phylum Actinobacteria (21). In long-term IBD, Yilmaz et al. defined distinct networks of taxa associations within intestinal biopsies of patients with CD and UC and reported that disturbances in an association network containing taxa of Lachnospiraceae and Ruminococcaceae families, typically producing short-chain acids, characterize frequently relapsing disease and poor response to treatment with anti-TNF-α therapeutic antibodies (22).

The abundance of sulfate-reducing bacteria (SRB), which produce hydrogen sulfide (H_2_S), in IBD has also been reported (23). The produced H_2_S is toxic to mucosal cells and inhibits the absorption of butyrate, induces cell overgrowth, and inhibits bacterial phagocytosis and killing (24); H_2_S-producing SRB is thus suspected to be involved in the pathogen of IBD. This report found a decrease in the *Clostridium* IXa and IV groups. In patients with active UC, the number of SRB was observed to be increased compared with those in patients with UC in remission and healthy controls (25–27). Various types of bacteria are included among SRB, including *Fusobacterium, Proteus, Campylobacter, Pseudomonas*, and *Salmonella* (28). Some studies point to the possibility that the dysbiosis of gut fungal microbiota, in addition to that of gut bacterial microbiota, may play a part in lesion development in IBD (29, 30).

### 2.3. Suspected causative bacteria of UC

Historically, numerous bacteria have been studied as the primary UC cause candidates. The major reports on those studies are summarized in Table 4 (23, 31–33). Of note, in many of those studies, bacteria were isolated and identified using stool cultures. Since infection is commonly initiated by the adherence of bacteria to host cell surfaces, the analysis of cultured mucosal bacteria is of greater value than the analysis of cultured stool bacteria; therefore, the use of mucosal cultures for the isolation and identification of bacteria is desired and preferred.

**TABLE 4:** Suspected causative bacteria for UC.

References	Causative bacteria	Specimens tested
Roediger et al. (23)	Sulfate-reducing bacteria	Mucosa
Matsuda et al. (31)	*Bacteroides vulgatus*	Mucosa
Ohkusa et al. (32)	*Fusobacterium varium*	Mucosa
Swidsinski et al. (33)	*Bacteroides, Enterobacteriaceae*	Mucosa

**FIGURE 2:**
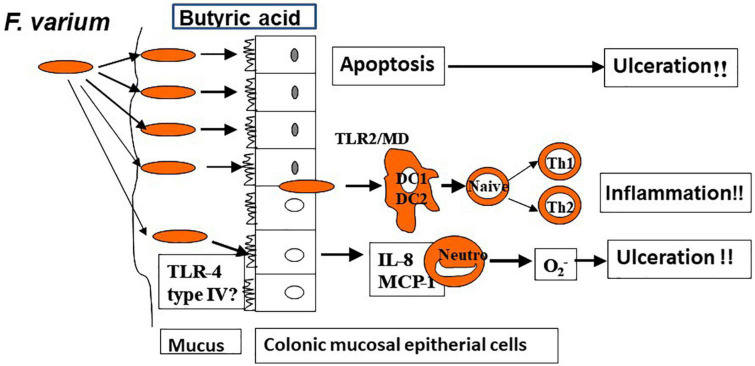
Colonic ulceration and inflammation by *Fusobacterium varium* (hypothesis).

In a prior study in which the author and associates closely examined surgically excised mucosal lesion samples from patients with UC, we found bacilli adherent to the lesions as well as mucosal invasion at ulcerated sites (34). We then isolated and identified *Fusobacterium varium* from the inflamed mucosa of patients with UC. The detection rate for these bacteria was significantly higher in patients with UC than in those with CD, ischemic enteritis, or colonic adenoma or in healthy controls. Moreover, the serum antibody titer against *F. varium* was significantly higher in samples from patients with UC. In addition, immunohistochemical staining of inflamed mucosal samples detected *F. varium* in a larger proportion of UC samples than other disorder samples or healthy control samples (32). Despite the absence of verotoxin genes, *F. varium* cells demonstrated cytotoxicity to Vero cells and were tested for the origin of the toxin. We found that the butyrate produced by *F. varium* was toxic to Vero cells and that enemas of this butyrate induced apoptotic changes and UC-like lesions in mice (35). It was reported that butyrate in the human colonic epithelium is an energy source but induces apoptosis (36). As an end product of dental plaque metabolism, butyrate is involved in the etiology of periodontal disease (37), whereas in the pediatric field, a butyrate-producing bacterium is reported to cause neonatal necrotizing enterocolitis (38). These reports support our hypothesis: *F. varium* bacteria adhere to or invade the colonic mucosa and generate butyrate, which induces ulceration in the colon. We also confirmed that such colonic mucosal adherent or invading bacteria markedly promoted the release of pro-inflammatory cytokines, such as IL-8 and tumor necrosis factor-α (TNF-α), from the mucosa (39). Moreover, our whole-genome sequencing of *F. varium* has revealed their high pathogenicity: they have the type IV secretion system found in *Helicobacter pylori* and the type V secretion system found in *Yersinia* and *Neisseria gonorrheae*, and they carry a large number of adhesins, which induce mucosal adherence, i.e., a basic property of pathogens (40). The above findings indicate that *F. varium* may elicit inflammation, ulceration, and, eventually, UC (Figure 2). Recently, increases in *Fusobacterium* group members have been reported in patients with UC after undergoing pouch surgery for pouchitis (41, 42). Another recent study found that patients with UC who did not achieve remission with fecal microbiota transplantation (FMT) had an enrichment of a *Fusobacterium* species (43).

We later attempted to reduce the load of *F. varium* in patients with UC positive for the antibody of this species using a combination therapy consisting of three antibiotics (amoxicillin, tetracycline, and metronidazole) to which *F. varium* is susceptible; improvement was achieved in UC symptoms and endoscopic and histological findings (44). In a double-blind, placebo-controlled, multicenter study of more than 200 patients, the aforementioned triple antibiotic regimen significantly exceeded the placebo in improvement and remission rates (45). The above findings indicate that *F. varium* is likely involved in the etiology of UC.

### 2.4. Suspected causative bacteria for CD

Of the several potential causes of CD reported to date (Table 5), the most notable is *Mycobacterial paratuberculosis* (currently classified as *Mycobacterium avium subspecies paratuberculosis*), reported by Chiodini et al. (46). *M. paratuberculosis* is known as a pathogen of Johne’s disease, contagious chronic enteritis in domestic animals with symptoms such as diarrhea and feces mixed with mucus and/or blood. This atypical acid-fast bacterium grows very slowly and is, therefore, very difficult to culture and identify in a conventional manner. For the first time, Chiodini et al. (46) succeeded in isolating the bacterium from patients with CD and culturing it. By employing a polymerase chain reaction (PCR)-based method, Sanderson et al. (47) detected a DNA element specific to *M. paratuberculosis* (i.e., IS900) in 65% of the CD patient tissue samples tested. Ryan et al. (48) used PCR to examine DNA extracted from laser capture microdissection-isolated granuloma tissue specimens and detected IS900 in 40% of the specimens from the studied patients with CD. Using culture and PCR, Naser et al. (49) surprisingly confirmed the presence of viable *M. paratuberculosis* in peripheral blood in a higher proportion of CD patient samples than in control samples. The role of *M. paratuberculosis* in the etiology of CD is still being actively debated today. In a study of rifaximin, this anti-*M. paratuberculosis* antibiotic was shown to be more effective than a placebo in inducing CD remission (50).

**TABLE 5:** Suspected causative bacteria for CD.

References	Causative bacteria	Specimens tested
Chiodini et al. (46)	*Mycobacterium paratuberculosis*	Mucosa, lymph node
Darfeuille-Michaud et al. (51)	Adherent *E. coli*	Mucosa
Willing	*Ruminococcus gnavus*	Mucosa
Strauss et al. (52)	*Fusobacterium nucleatum*	Mucosa
Atarashi et al. (53)	*Klebsiella pneumoniae*	Cecal fluid

Adherent-invasive *E. coli* is considered to be another potential cause of CD, as reported by Darfeuille-Michaud et al. (51). They cultured surgically resected ileal mucosa samples from patients with CD and detected α-hemolysin-producing adherent-invasive *E. coli* strains in many of the lesions evaluated (active lesions, 84.6%; non-active lesions, 78.9%; control, 33%). Recently, the presence of *Fusobacterium nucleatum* in CD-affected intestinal mucosa has also been reported. Strauss et al. (52) detected strains of this species in 10 (58.8%) of 17 patients with CD, all of which were cytoinvasive. Atarashi et al. (53) demonstrated that *Klebsiella pneumoniae* isolated from the cecal fluid of patients with CD upon colonizing the mouse gut mucosa strongly induced interferon gamma (IFN-γ)-producing CD4 + T helper cells, indicating the possibility for this species to cause intestinal inflammation and exacerbate CD. Recently, Federici et al. transferred clinical IBD-associated *K. pneumoniae* into germ-free mice and found that colonized mice enhanced intestinal inflammation. In addition, they showed that a lytic five-phage combination targeting *K. pneumoniae* enables effective *K. pneumoniae* suppression in colitis-prone mice (54).

## 3. IBS and intestinal bacteria

In the 1960s, when the first modern concept of IBS was proposed, it was already known that IBS could develop following infectious enteritis (55). In Balsari et al. (56) reported, as revealed by stool culture analysis, a decrease in bifidobacteria, lactobacilli, and anaerobic bacteria in patients with IBS compared with healthy individuals. Their findings drew little attention, possibly because IBS was largely believed to be attributable to GI dysfunction. Later, in Pimentel et al. (57) found small intestinal bacterial overgrowth (SIBO) in a large proportion of the studied patients with diarrhea-predominant IBS. The symptoms of this type of IBS were shown to be improved by treatment with antibiotics (such as rifaximin) or probiotics (such as VSL#3); the relationships between intestinal bacteria and IBS have thus come to garner increasing attention (58). According to the findings from a systematic review, increases in the families Enterobacteriaceae and Lactobacillaceae and the genus *Bacteroides* and decreases in the genera *Bifidobacterium* and *Faecalibacterium* were demonstrated in patients with IBS (59).

With respect to constipation-predominant IBS, an association of methanogenic bacteria is indicated by the increased methane concentration in the stool of patients with constipation (60). The primary methanogenic bacterial species was *Methanobrevibacter smithii*. One study reported that a neomycin and rifaximin combination therapy targeting this species resulted in an improvement in clinical symptoms as well as a decrease in methane in the stool (61).

Irritable bowel syndrome, especially IBS following infectious enteritis, is associated with decreased occludin at tight junctions and increased colonic mucosal permeability. This results from an increase in serine protease due to gut microbiota dysbiosis. One recent study reported an improvement in mucosal permeability by treatment with a serine protease inhibitor (62). Dysbiosis and leaky gut are deemed causative factors for IBS. In diarrhea-predominant IBS, bile acid malabsorption is observed. It has been reported that this inhibition of absorption is strongly linked to the gut microbiota dysbiosis-associated changes in the fecal metabolome consisting of stool metabolites (63).

## 4. Colorectal cancer and intestinal bacteria

Colorectal cancer (CRC)-associated intestinal bacteria reported to date based on bacterial culture analysis include pathogenic strains of *E. coli*, enterotoxigenic strains of *Bacteroides fragilis*, and *Streptococcus bovis* (64). In a recent meta-analysis of *S. bovis* based on stool culture, patients with CRC were found to have a higher incidence of this species in their feces, with an odds ratio of 2.52 (95% confidence interval, 1.14–5.58). This finding suggests a significant association of *S. bovis* with CRC (65). Conversely, in one study, bacterial genomic analysis detected a higher prevalence of genetic polymorphism for the *Clostridium coccoides* and *Clostridium leptum* groups in the stool samples of CRC patients than in those of healthy controls, and in another study, increased coriobacteria and decreased enterobacteria were observed in CRC tissue compared with adjacent non-malignant mucosa (64). Ahn et al. (66) reported the highest enrichment of *Fusobacterium*, followed by *Porphyromonas*, in patients with CRC. One study found that *Fusobacterium* was significantly abundant in CRC tissue and that fluorescence *in situ* hybridization detected an enrichment of the bacteria of this genus in CRC compared with non-neoplastic mucosa (67). Another study reported that a *Fusobacterium* isolate was obtained from a cancer tissue specimen (68). These findings have made *Fusobacterium* a high-profile factor worldwide, similar to *H. pylori*, as a potential cause of CRC. Essentially, *F. nucleatum* cultured from cancer tissue promotes BRAF mutations and microsatellite instability, increasing the risk of CRC in an experimental setting. The reduction in this species with antibiotic treatment was reported to inhibit tumor growth in number and size (69). With regard to Japanese individuals, Yachida et al. (70) reported that the whole metagenomic analysis of stool samples showed an increase in *Atopobium parvulum* in colonic adenomas and a significant elevation in both *F. nucleatum* and *Actinomyces odontolyticus* in intramucosal carcinomas, a more advanced stage of the tumor. Thomas et al. performed a meta-analysis of five publicly available data sets and two new cohorts and validated the findings on two additional cohorts, considering a total of 969 fecal metagenomes. Unlike microbiome shifts associated with GI syndromes, the gut microbiome in CRC showed reproducibly higher richness than controls (*P* < 0.01), partially due to expansions of species typically derived from the oral cavity. They found that *Peptostreptococcus stomatis* was the species with the highest average rank in CRC (71).

In our 16S rRNA metagenomic analysis of aspirates from colorectal adenoma, which is a precancerous lesion, *F. nucleatum* was detected, but not in significant abundance compared with normal colorectal tissue. Conversely, *F. varium*, another species belonging to Fusobacteria, was significantly enriched in adenoma samples compared with normal samples (72). A Hong Kong study also reported that an abundance of *F. varium*, but not *F. nucleatum*, was detected by shotgun whole metagenomic analysis in patients with CRC (73). In a large study where more than 50,000 male subjects were evaluated in terms of their diet patterns and fecal microbiota over 26 years, an increase in H_2_S-producing SRB in stools was associated with an increased risk of the distal colon and rectal cancers (74). SRB, which includes Fusobacteria, are well-known periodontal pathogens; oral bacteria are implicated in CRC. Recently, Bertocchi et al. demonstrated the critical role of the gut vascular barrier (GVB) in the hematogenous route of liver CRC metastases. They link GVB impairment with bacterial translocation into the liver, the formation of a premetastatic niche, and tumor cell seeding. We report that tumor-resident bacteria *E. coli* disrupt the GVB and antibiotic treatment hampers metastases formation, preventing GVB disruption (75). They proposed that targeting tumor-associated bacteria might be a new strategy to counteract liver metastases.

## 5. Esophageal cancer and intestinal bacteria

Esophageal cancers are roughly divided into esophageal squamous cell cancer (ESCC) and esophageal adenocarcinoma (EAC); ESCC is predominant in Asia and Africa, whereas EAC is more prevalent in Europe and the United States (USA). Yamamura et al. (76) of Japan detected *F. nucleatum* in 23% (74/325) of resected esophageal cancer tissue specimens. A study by Gao et al. (77) detected *Porphyromonas gingivalis* in 61% of the ESCC tissue samples studied. Peters et al. (78) performed a 16S rRNA meta-analysis on oral bacteria from patients with esophageal cancer and found that the periodontal pathogen *P. gingivalis* was abundant in ESCC samples and *Tannerella forsythia* in EAC samples. In a recent study, periodontal pathogens in dental plaque and saliva samples from 61 patients with ESCC were subjected to a real-time PCR assay, and the results were subjected to logistic regression analysis and found *T. forsythia* and *Streptococcus anginosus* in dental plaque, *Aggregatibacter actinomycetemcomitans* in saliva, and a drinking habit was found to be associated with a significantly higher ESCC risk, but *F. nucleatum* was not significantly associated with ESCC risk (79). Since most of the above bacteria detected in esophageal cancer live in the oral cavity, it is suspected that oral bacteria are involved in esophageal cancers.

## 6. Non-steroidal anti-inflammatory drug-induced enteritis and intestinal bacteria

Non-steroidal anti-inflammatory drugs (NSAIDs), well-known inducers of the stomach and duodenal ulcers, also induce small intestinal ulcers, but this is not broadly known. The increasing prevalence of capsule endoscopy and enteroscopy for the small intestine has shed light on the high ulceration rate (over 50%) following NSAID treatment, which has emerged as a clinical problem that causes intestinal bleeding. In Kent and associates demonstrated that small intestinal ulcerations developed in almost 100% of indomethacin-treated rats and that antibiotic combination treatment with neomycin, polymyxin B, and bacitracin reduced the severity of ulcers (80). Culture analysis revealed a significant increase in the numbers of *E. coli, Bacteroides*, and clostridia at the time of ulceration. Antibiotic treatment reduced the numbers of these bacteria to near-normal levels. These findings suggested the involvement of the above intestinal bacteria in small intestinal ulcerations. Furthermore, NSAID treatment was not shown to result in small intestinal ulceration in rats when bile was excluded by bile duct ligation, suggesting a potential role of bile acid in small intestinal ulcer formation (81). Robert and Asano (82) reported that indomethacin did not induce small intestinal ulcers in germ-free rats. In a Japanese study by Uejima et al. (83) in following cyclooxygenase-2 inhibitor treatment, no small intestinal ulcers were observed in germ-free rats, whereas ulceration was observed in 57–71% of specific pathogen-free rats. Small intestinal ulcers did not develop in specific pathogen-free rats after cotreatment with three antibiotics (neomycin, streptomycin, and bacitracin). Culture analysis revealed an increase in Gram-negative bacilli (e.g., *E. coli, Klebsiella, Proteus*, and *Bacteroides*) in both the feces and lesions of the animals, whereas these bacteria were markedly decreased by antibiotic treatment. The authors concluded that their findings suggested an association of Gram-negative bacilli with ulcer formation. As indicated by the above data, intestinal bacteria are deemed to be responsible for NSAID-induced ulceration in the small intestine. Another study found that probiotic treatment using *Bifidobacterium breve* reduced both the number and size of aspirin-induced small intestinal ulcers compared with placebo (84).

## 7. Alcoholic steatohepatitis (ASH), NASH, and intestinal bacteria

**FIGURE 3:**
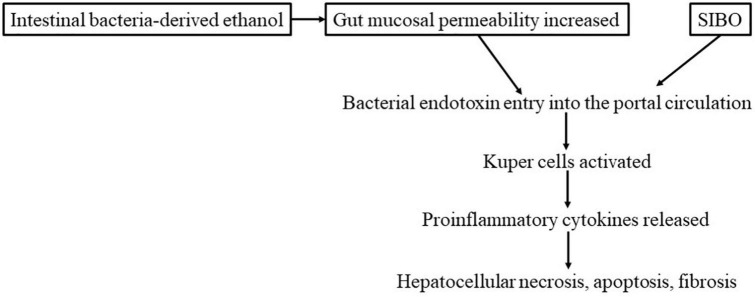
Development and exacerbation of NASH by intestinal bacteria (hypothesis).

The likely mechanism of the development of ASH is postulated to be as follows: alcohol increases mucosal permeability. This increased mucosal permeability allows large quantities of endotoxins (lipopolysaccharide, LPS) to leak into the portal venous circulation. LPS then activates Kupffer cells in the liver, enhancing the release of pro-inflammatory cytokines, including TNF-α, by these cells. Such cytokines contribute to hepatocellular necrosis, apoptosis, and fibrosis (Figure 3). Some findings have suggested that hepatic necrosis, apoptosis, and fibrosis in NASH may be caused in a similar manner by factors such as intestinal bacteria-derived alcohol (85) and intestinal bacterial overgrowth (86). With regard to genetic elements, it is known that in both ASH and NASH patients, the expression of the LPS receptor CD14 in Kupffer cells is elevated, increasing their susceptibility to LPS (87). Treatment with probiotics or FMT has been reported to inhibit the development of both ASH and NASH and improve the lesions of these conditions (86). This finding indicates a close association of not only insulin resistance but also intestinal bacteria-derived LPS in the pathogenesis of ASH and NASH. A recent study by Sookoian et al. (88) detected intestinal bacteria, albeit in small amounts, in NASH liver tissue samples; among them, Bacteroidetes, Firmicutes, and Proteobacteria were overrepresented, whereas the abundance of Lachnospiraceae was decreased.

In an interesting animal model study, mice in which NASH was induced by feeding with a methionine-choline-deficient diet were cohoused with wild-type mice of the same strain as the NASH model mice; the wild-type mice also developed NASH (89). The pyrosequencing of the fecal microbiota from those mice demonstrated an increased representation of *Prevotella*. A study by Zhu et al. (90) also reported an increase in *Prevotella* abundance in children with NASH compared with healthy controls (91). However, in another study, adult patients with NASH were found to have a lower percentage of Bacteroidetes (the phylum to which the genus *Prevotella* belongs) in their total bacterial counts (92). Consistency in findings is yet to be achieved. Increases in *Prevotella* have also been reported in samples from patients with alcoholic cirrhosis (93). There is a report that SIBO is present in 50% of patients with NASH, and another report states that SIBO is common in patients with liver cirrhosis (LC) (91).

Naturally, alcohol abstinence is a primary strategy in the treatment of ASH; other options include treatments targeting intestinal bacterial dysbiosis. An overabundance of LPS, a potential cause of ASH, is induced by Gram-negative bacteria, antibiotics (such as rifaximin) or probiotics (such as lactobacilli and bifidobacteria) are thus administered to decrease Gram-negative bacteria and thereby reduce LPS, with a certain degree of therapeutic success, such as improved hepatic function (94). NASH is mainly treated by weight loss and with the aforementioned antibiotic, probiotic, prebiotic, synbiotic, and fiber diet approach for the same reason as in the case of ASH, yielding similar efficacy in NASH (95–99).

## 8. LC, hepatic encephalopathy, and intestinal bacteria

With regard to intestinal bacterial dysbiosis in LC, fecal 16S metagenomic sequencing revealed the following: an enrichment of Proteobacteria and Fusobacteria and a reduced proportion of Bacteroidetes in one study (100) and an increase in Staphylococcaceae, Enterococcaceae, and Enterobacteriaceae, and a decrease in *Clostridium* XIV, Lachnospiraceae, Ruminococcaceae, and Rikenellaceae in another study (101). Enterococcaceae and Enterobacteriaceae are families of the phylum Proteobacteria; increases in the number of Proteobacteria are almost certain to occur in LC. Furthermore, Sung et al. (102) found a decreased abundance of Bacteroidetes and increased abundances of Firmicutes, Proteobacteria, and Actinobacteria in decompensated LC with hepatic encephalopathy (HE) compared with the fecal microbiome in compensated LC. Of those findings, an increase in *Veillonella parvula* of the phylum Firmicutes was especially conspicuous. Their 1-year follow-up data demonstrated that HE recurrence was associated with increased abundances of *Clostridium* XI, *Bacteroides, Lactobacillus*, and *Clostridium sedis* and decreased abundances of *Alistipes, Bacteroides*, and *Phascolarctobacterium*. The author and associates performed metagenomic sequencing on blood samples obtained from 66 patients with LC and created blood microbial profiles, which demonstrated an increased abundance of Enterobacteriaceae and decreased abundances of *Akkermansia*, Rikenellaceae, and *Erysipelotrichales* compared with healthy control profiles (103). These findings are consistent with the results of the aforementioned studies (100–102). Intestinal bacteria were shown to pass through the intestinal mucosa and enter the blood circulation (bacterial translocation). Numerous bacterial species are assumed to be present in the blood of patients with LC (103), which possibly contributes to the development of LC complications, such as sepsis and spontaneous bacterial peritonitis. Bacterial translocation is closely related to various liver disease states; for example, the entry of bacterial toxins (such as endotoxins) and bacterial metabolites (such as ammonia and mercaptan) into the blood circulation is associated with the state of HE and other liver diseases (104). Thus, the above study results are helpful in planning therapeutic strategies, e.g., the use of antibacterial or probiotic agents in patients with LC. Essentially, the non-absorbable antibiotic rifaximin is effective in treating HE, with long-term effects demonstrated over extended periods (105). Lactulose is an oligosaccharide with a long history of therapeutic use for HE. This substance is deemed to exert its effect by improving gut dysbiosis as a prebiotic (106).

## 9. Primary sclerosing cholangitis and intestinal bacteria

Primary sclerosing cholangitis (PSC) is a chronic progressive inflammatory disease characterized by fibrous strictures of intrahepatic and extrahepatic bile ducts. In Japan, PSC is designated as an intractable disease. Fecal microbiota dysbiosis has been reported in PSC. In a patient study by Iwasawa et al. (107), 16S metagenomic analysis revealed an overrepresentation of the genus *Enterococcus* of the phylum Firmicutes and a decreased abundance of the members of the genus *Parabacteroides* in PSC patients compared with healthy controls; increased species of Enterococcus included *Streptococcus parasanguinis, Veillonella* sp., and *Enterococcus faecium*. Sabino et al. (108) of Belgium reported significantly increased abundances of *Fusobacterium* and *Lactobacillus* in addition to an overrepresentation of *Enterococcus* in PSC patient samples. Adopting a similar analysis method, Rühlemann et al. (109) of Germany detected an increase in the phylum Proteobacteria and the bile tolerant genus *Parabacteroides* in PSC. Liwinski et al. (110) analyzed bile specimens obtained from patients with PSC and found an increase in *E. faecalis*, which correlated with the increased concentrations of the secondary bile acid taurolithocholic acid. In an animal study, cholangitis was reproduced in gnotobiotic mice inoculated with *K. pneumoniae* that was isolated from PSC patient stool samples (111). Allegretti et al. (112) performed FMT, although in a small number of patients (*n* = 10), to evaluate its effectiveness on dysbiosis in PSC; an improvement in alkaline phosphatase levels, a measure of PSC lesion severity, was observed in three (30%) patients.

## 10. Pancreatic cancer and intestinal bacteria

In Michaud et al. (113) reported a statistically significant association of periodontal disease with pancreatic cancer risk, which prompted further studies on the relationship between periodontal disease and pancreatic cancer. Moreover, significantly increased abundances of periodontal disease pathogens (e.g., *P. gingivalis* and *A. actinomycetemcomitans*) in the oral cavity of patients with pancreatic cancer have been reported (114, 115). In a study by Mitsuhashi et al. (116), *Fusobacterium* species were detected in 25 (8.8%) of 283 pancreatic cancer tissue specimens. Geller et al. (117) reported that the most commonly identified species in their pancreatic cancer samples were those belonging to the class Gammaproteobacteria. Pushalkar et al. (118) detected bacterial DNA in significantly larger proportions of pancreatic cancer tissue specimens than healthy control specimens, with particularly high abundances of Proteobacteria, Bacteroidetes, and Firmicutes and the genera *Pseudomonas* and *Elizabethkingia*. In addition, in fecal samples from patients with pancreatic cancer, Pushalkar et al. (118) found increased abundances of Proteobacteria, Synergistetes, and Euryarchaeota, exhibiting dysbiosis. Recently, Kartal et al. reported that *Veillonella atypica, F. nucleatum/hwasookii*, and *Alloscardovia omnicolens* were enriched in the feces of patients with PDAC, whereas *Romboutsia timonensis, Faecalibacterium prausnitzii, Bacteroides coprocola*, and *Bifidobacterium bifidum* species clusters were depleted (119). As shown above, the bacterial invasion of pancreatic tissue is deemed responsible for the inflammation and immune system modifications that promote cancer growth. In contrast, there is a report that a higher tumoral microbial diversity is associated with longer term survival (120). The above are findings from solid pancreatic ductal adenocarcinomas, but similar observations have also been reported for pancreatic cysts. Enrichment of the oral bacteria *F. nucleatum* and *Granulicatella adiacens* was detected in the pancreatic cyst fluid of intraductal papillary mucinous neoplasms with high-grade dysplasia, which is a precursor to cystic pancreatic cancer (121).

## 11. Treatment for intestinal bacterial dysbiosis

Diseases associated with gut microbiota dysbiosis, leaky gut with increased mucosal permeability, and SIBO have been described. Treatments for such diseases include antibiotics and probiotics (e.g., lactobacilli and bifidobacteria), prebiotics (e.g., oligosaccharides), synbiotics (i.e., a combination of probiotics and prebiotics), and FMT. These long-standing traditional therapies are now provided with the aim of reducing causative bacterial groups and correcting gut dysbiosis, as gut microbiota dysbiosis has been acknowledged as one of the causes of each of the above diseases.

Antibiotics have actually been administered to HE (105), IBS (58), and UC (44), and their efficacy has been reported. In addition, probiotics, prebiotics, and synbiotics are therapeutic use for IBS (58), NSAID-induced enteritis (84), NASH (95), and HE (106).

### 11.1. FMT for antibiotic-induced enteritis, including clostridium difficile infection

In 1935, a new species of bacteria was named *Bacillus difficilis*, the species name was given because of its difficult anaerobic isolation from human feces. After 40 years, *B. difficilis* was renamed *Clostridium difficile* and identified as the cause of pseudomembranous colitis. This microorganism produces a toxin that secretes fluids and leads to the development of yellow-white plaques on the colonic mucosa. Essentially, microbial substitution resulting from antibiotic therapy allows the overgrowth of this resident microorganism in the gut microbiota, leading to the generation of toxicity. In our experience, *C. difficile*, being susceptible to vancomycin and metronidazole, can usually be removed by either of these antibiotics before inducing intractable conditions (122).

In Europe and the USA, however, recurrent *C. difficile* infection (CDI) and CDI resistant to the above two antibiotics have been observed for some time, and to make matters worse, CDIs have been on the rise in recent years, looming large as a serious clinical problem. For such refractory CDIs, FMT is considered the only effective therapy, and numerous case reports on FMT-treated patients have been published to date. A systematic review by Sha et al. (123) is available, covering an extensive range of these reports, including abstracts presented at academic meetings. The first modern FMT treatment of CDI was performed in by Eiseman et al. (124). This FMT treatment was given as one to three fecal enemas in four patients with recurrent CDI and resulted in symptom improvement and the clearance of the causative pathogen in all patients. No adverse events were observed. In FMT-treated patients who followed from then on, the success rates remained high, ranging from 79.7 to 100%, with few adverse events reported. The main administration method was initially retention of enemas *via* a rectal tube, which was then replaced by duodenal infusion *via* a nasogastric tube or gastroscopy, and recently, FMT administration by colonoscopy has become increasingly common. The dose used varies from patient to patient, but it seems that larger doses can be delivered *via* the colonic route than the duodenal route. Prior to FMT, vancomycin or metronidazole is administered in many patients. The aim of this pretreatment may be to reduce, if not eradicate, *C. difficile* to improve the microbiota composition rich in this microorganism to the greatest extent possible. The report by Van Nood et al. (125), which is the only controlled FMT study, surprised healthcare professionals worldwide.

The comparison between vancomycin alone and FMT resulted in significantly higher CDI resolution rates for FMT (81.3–91.8%) than for the vancomycin regimens tested (23.1–30.8%), demonstrating the superiority of FMT to this antibiotic (125). Recently, Baunwall et al. reported that oral vancomycin therapy followed by FMT for the first or second CDI resulted in a significantly higher cure rate than that of a placebo (126). Kelly et al. (127) conducted FMT in 80 immunocompromised (due to HIV, immunosuppressive therapy, or other reasons) patients with CDI and achieved a CDI cure rate of 89%. However, 12 (15%) patients had serious adverse events. Caution for adverse events is required in the treatment of immunocompromised patients with FMT. 16S rRNA analysis-based assessments of post-FMT changes in the microbiota conducted by Van Nood et al. (125), Dutta et al. (128), and Seekatz et al. (129) all revealed increased microbial diversity, similar to the donor profile, following FMT. This result indicates the successful transplantation of a healthy donor’s gut microbiota into the patient. Dutta et al. (128) found significantly increasing proportions of the family Lachnospiraceae and decreasing proportions of Enterobacteriaceae, whereas Van Nood et al. (125) and Seekatz et al. (129) reported decreases in the phylum Proteobacteria, especially an Enterobacteriaceae member at the genus level, as well as increases in the phylum Bacteroidetes and phylum Firmicutes, including *Clostridium* clusters IV and XIVa. These findings suggest that donor gut microbiota transplantation *via* FMT can achieve the successful elimination of *C. difficile* and the resolution of CDI symptoms.

### 11.2. FMT treatment for IBD

Fecal microbiota transplantation treatment of IBD was first reported in by Bennet and Brinkman (130). One patient underwent antibiotic treatment for intestinal sterilization, followed by an FMT enema. A single FMT achieved remission (130). Subsequently, randomized controlled trials (RCTs) were conducted in a double-blind manner for the objective evaluation of FMT treatment of IBD (131–134). Paramsothy et al. (133) performed intensive-dosing FMT by colonoscopy 5 days per week for 8 consecutive weeks; the remission rate was significantly higher for the FMT group (27%, 11/41 patients) than for the placebo group (8%, 3/40 patients). In these four RCTs, FMT was found to be effective in three (131, 133, 135), except the trial by Rossen et al. (132), which was a negative trial. With improved fecal delivery techniques, the efficacy rates have been increasing recently. Ishikawa et al. (135) of Japan assessed a treatment method that applied a bone marrow transplant method combined with a multiple antibiotic regimen in patients with UC who responded well to FMT enemas following multiple antibiotic pretreatment for intestinal sterilization, with a clinical response rate of 82.3% and a remission rate of 53.0% at 4 weeks. Furthermore, in another study of FMT plus the same multiple antibiotic pretreatment as above, Ishikawa et al. (136) observed a recovery of bacterial diversity with increased Bacteroidetes species in most responders, resulting in bacterial profiles that were similar to those of donors. Recently, two studies conducted in RCTs of similar multiple antibiotic pretreatment and FMT were performed by oral capsules (137) or lyophilized capsules (138) for UC and reported their effectiveness. However, the clinical efficacy of FMT treatment of UC remains elusive despite favorable outcomes, such as the above, due to the variability in treatment modalities studied thus far and small patient sample sizes. Sarbagili Shabat et al. evaluated whether the integration of novel UC exclusion diets (UCED) for patients with UC, in addition to FMT, could increase the FMT remission rate in refractory UC. The results of the study were that UCED alone appeared to achieve higher clinical remission and mucosal healing than single-donor FT with or without diet (139). Karjalainen et al. reported an RCT study in which the safety profile of FMT was good, but FMT was not effective in the treatment of chronic pouchitis (140).

With respect to FMT treatment of CD, FMT by retention enema in a 31-year-old male patient was reported in by Borody et al. (141), and this treatment was drastically effective, achieving clinical remission with no adverse events. Paramsothy et al. (142) demonstrated remission rates of 50.6% (42/83 patients) and 52.0% in patients with CD receiving FMT treatment included in their meta-analysis of cohort studies. Li et al. (143) of China performed FMT by endoscopy in 69 patients with CD by infusing a 150-ml microbiota suspension from the duodenum below the papilla of Vater in the anal direction, and favorable outcomes were achieved, with an improvement rate of 62.3% (43/69 patients) and a remission rate of 43.5% (30/69) at 4 weeks post-FMT. Notwithstanding the above, the efficacy of FMT treatment of CD is yet to be established due to the limited number of patients studied to date and the absence of RCTs. UC and CD both comprise diverse disease states accompanied by genetic polymorphic variations.

It is not clear which states are or are not dysbiosis related. Cases of IBD that benefit from FMT may thus be limited.

### 11.3. FMT treatment of IBS

In Borody et al. (141) performed FMT by retention enema in a 21-year-old female patient with diarrhea-predominant IBS and reported good outcomes with symptom resolution and a cure. Six RCTs followed (144–149) with mixed results depending on the FMT treatment methods used, including non-response to oral FMT capsule therapy (two studies), response to duodenal or jejunal delivery by nasogastroscopy (two studies), response to cecal delivery by endoscopy (one study), and non-response to the same modality (one study). Thus, the efficacy of FMT treatment of IBS is not clear.

### 11.4. Adverse events of FMT treatment

According to the FMT treatment, adverse event data compiled by Lai et al. (150) from 4,493 patients in 35 studies were published up to mid-2018 and the serious adverse events reported were as follows: death (0.13%), aspiration pneumonia (0.16%), and intestinal perforation and sepsis (0.07% each). The most common events included diarrhea (13%) and abdominal distension (11%). One case of death from multidrug-resistant *E. coli* infection among FMT-treated patients was reported in DeFilipp et al. (151), raising a warning against the use of FMT without solid reasoning. FMT therapy is not without weaknesses, which are said to include the inability to eliminate unknown viruses. The elucidation of dysbiosis at the bacterial species level and, based on the uncovered data, the development of pharmaceuticals comprising numerous probiotic intestinal bacteria as alternatives to FMT are awaited.

## 12. Conclusion and future perspectives

The detection of intestinal bacteria in tissue samples of pancreatic and other cancers indicates an association of microbiota with cancer development and proliferation and, further, with patient responses to anticancer drugs. Various diseases are assumed to be caused by the loss of microbiota diversity and the resulting dysbiosis. Increases and decreases in bacterial populations occur in diseased states (Table 6), and causative pathogens are probably among those that increase. We should not perceive dysbiosis as a single cause but look further into it and identify specific causative bacteria or bacterial groups. Currently, microbiota analysis systems are advancing from 16S metagenomic sequencing to whole-genome shotgun sequencing, which allows comprehensive sampling of all genes in given specimens, along with the establishment of computer analysis systems. Against such a backdrop, further exploration of causative pathogens is expected to proceed. Revolutionary therapies for the diseases discussed herein may then come into sight. We look forward to seeing what lies ahead.

**TABLE 6:** More and less abundant bacteria in GI disorders.

Bacteria	Diseases				
**More abundant**					
Bacteroidetes	NASH	PC			
Bacteroides	UC	IBS	CRC	NSAID enteritis	
*Bacteroides vulgatus*	UC				
*Porphyromonas gingivalis*	ESCC				
*Tannerella forsythia*	EAC				
Proteobacteria	NASH	HE	PC		
Enterobacteriaceae	UC				
Adherent *E. coli*	CD				
*Klebsiella pneumoniae*	CD				
*Enterobacteriaceae*	IBS	LC			
*E. coli*	CRC	NSAID enteritis			
Firmicutes	NASH	HE	PC		
*Ruminococcus gnavus*	CD				
*Lactobacillaceae*	IBS				
*Streptococcus bovis*	CRC				
*Clostridium coccoides*	CRC				
*Clostridium leptum*	CRC				
*Peptostreptococcus stomatis*	CRC				
*Clostridia*	NSAID enteritis				
*Enterococcaceae*	LC				
*Staphylococcaceae*	LC				
*Enterococcus*	PSC				
*Lactobacillus*	PSC				
*Granulicatella adiacens*	PC				
Actinobacteria	HE				
*Mycobacterium paratuberculosis*	CD				
*Actinomyces odontolyticus*	CRC				
**Fusobacteriota**					
*Fusobacterium*	PSC				
*Fusobacterium nucleatum*	CD	CRC	CRA	ESCC	PC
*Fusobacterium varium*	UC	CRA			
**Archaea**					
*Methanobrevibacter smithii*	IBS-C				
**Less abundant**					
*Lactobacillus*	UC	CD	IBS		
*Faecalibacterium prausnitzii*	UC	CD	IBS		
*Roseburia hominis*	UC	CD			
*Clostridium* IXa and IV groups	UC	CD			
*Bifidobacterium*	UC	CD	IBS		
Enterobacteria	CRC				
Lachnospiraceae	NASH				
Bacteroidetes	HE				
*Bacteroides*	HE				
*Alistipes*	HE				
*Phascolarctobacterium*	HE				
*Akkermansia*	LC				
*Rikenellaceae*	LC				
*Erysipelotrichales*	LC				
*Romboutsia timonensi*	PC				
*Faecalibacterium prausnitzii*	PC				
*Bacteroides coprocola*	PC				
*Bifidobacterium bifidum*	PC				

NASH, non-alcoholic steatohepatitis; PC, pancreatic cancer; UC, ulcerative colitis; IBS, irritable bowel syndrome; IBS-C, IBS constipation type; CRC, colorectal cancer; HE, hepatic encephalopathy; LC, liver cirrhosis; ESCC, esophageal squamous cell cancer; EAC, esophageal adenocarcinoma; PC, pancreatic cancer; CD, Crohn’s disease; NSAID, non-steroidal anti-inflammatory drugs.

## Author contributions

TO and NS wrote and edited the manuscript. TO created the figures and critically revised the manuscript. YN gathered the documents and created the tables. All authors read and approved the final manuscript for publication.
